# Effect of interprofessional education on oral assessment performance of nursing students

**DOI:** 10.1002/cre2.248

**Published:** 2019-10-10

**Authors:** Satoru Haresaku, Maki Miyoshi, Keiko Kubota, Hisae Aoki, Emi Kajiwara, Mayumi Monji, Toru Naito

**Affiliations:** ^1^ Department of Nursing Fukuoka Nursing College Fukuoka Japan; ^2^ Section of Geriatric Dentistry, Department of General Dentistry Fukuoka Dental College Fukuoka Japan

**Keywords:** nursing education, nursing student, oral assessment, oral health care

## Abstract

**Objectives:**

The purpose of this study was to investigate the effect of interprofessional educational programmes on the improvement of nursing students' oral assessment performances by comparing their attitudes, confidence, abilities, and self‐performance before and after the education.

**Materials and methods:**

The subjects included 112 first‐year nursing students in a Japanese nursing school. They participated in the oral assessment educational programmes, which were supported by dentists and introduced into the 1‐year curriculum. The first programme was a 1.5‐hr lecture with a self‐oral assessment training in May 2018, and the second was a 1‐hr oral assessment training in October 2018. The questionnaire surveys investigating nursing students' attitudes, confidence, and self‐performance regarding oral assessment and the tests measuring their oral assessment abilities were conducted before and after the programmes. The total scores on the tests were 0–9 points.

**Results:**

A total of 101 (90.2%) nursing students responded to all the questionnaires and tests. Their attitudes and confidence regarding oral assessment were significantly improved after the programmes. The total average scores on the tests were significantly increased from 6.8 points at baseline to 7.9 points after the programmes. The percentage of their performance of self‐oral assessment every day significantly increased from 15.8% at baseline to 32.7% after the programmes.

**Conclusions:**

These results suggested that the educational programme might be effective in improving not only the students' attitudes and confidence regarding oral assessment but also their oral assessment abilities and self‐oral assessment performance. Therefore, future programmes must focus on training them to improve oral health care referrals.

## 1 INTRODUCTION

Recent studies have shown that periodontal disease is an independent risk factor for cardiovascular disease (Humphrey, Fu, Buckley, Freeman, & Helfand, 2008), diabetes (Casanova, Hughes, & Preshaw, 2014), and preterm, low birth weight (Ide & Papapanou, 2013). Oral health care is effective for the prevention of aspiration pneumonia and ventilator‐associated pneumonia (Hua et al., 2016; Soutome et al., 2017; van der Maarel‐Wierink, Vanobbergen, Bronkhorst, Schols, & de Baat, 2013). In addition, a previous study reported that professional oral health care reduced oral mucositis pain in patients treated with chemotherapy (Kubota et al., 2017). Therefore, appropriate oral health care services should be provided for patients in hospitals to prevent not only dental diseases but also systemic diseases.

However, oral health check‐ups by dentists are difficult in hospitalized patients when there is no dental department in the hospital. A previous study reported that the inability to perform regular oral health check‐ups for inpatients was a major barrier to providing appropriate dental care to these patients (Nitschke, Müller, & Hopfenmüller, 2001).

To resolve this problem, the nurses who generally provide inpatient care may play an important role in performing oral assessments and appropriate referrals. Some studies reported nurses' oral assessment ability and developed tools for oral assessment (Andersson, Hallberg, & Renvert, 2002; Aoki et al., 2018; Chalmers, King, Spencer, Wright, & Carter, 2005; Eilers, Berger, & Petersen, 1988; Tsukada, Ito, Stegaroiu, Shibata, & Ohuchi, 2017). Those studies showed that the validity and reliability of the nurses' oral assessments with the usage of these tools were high. Therefore, it is suggested that oral assessments performed by nurses are good indicators of oral problems and enable them to make dental referrals to provide oral health services for patients.

However, a previous study of oral assessments performed by nurses in a hospital reported that only 51.7% of 143 nurses had performed oral assessments for elderly patients and that only one of them had used an oral assessment tool (Haresaku et al., 2018a). These results may indicate that applications of oral assessment tools are not currently as developed in Japan. Therefore, education to develop oral assessment performance with oral assessment tools should be provided to nurses in order to promote their dental referrals.

Several studies of nurses and dental professionals have described the effects of interprofessional education on students (Cooper et al., 2017; Estes et al., 2018; Golinveaux et al., 2013). There were also some studies regarding the effects of interprofessional education that specialized in improving nurses' oral assessment performance (Andersson et al., 2002; Aoki et al., 2018; Chalmers et al., 2005; Coan, Wijesuriya, & Seibert, 2019; Eilers et al., 1988; Haresaku et al., 2018b; Munoz, Touger‐Decker, Byham‐Gray, & Maillet, 2009; Tsukada et al., 2017). In addition, a previous study of nursing students reported that their confidence in oral assessment performance was improved after the oral assessment interprofessional education programmes (Estes et al., 2018). However, there were no studies regarding the effect of interprofessional education programmes that specialized in improving nursing students' abilities to perform oral assessments and their self‐oral assessment performance.

The purpose of this study was to investigate the effects of interprofessional educational programmes on the improvement of nursing students' oral assessment performance by comparing their attitudes, confidence, abilities, and self‐performance before and after the education.

## 2 METHODS

### 2.1 Study design and study sample

This study was a before–after survey of first‐year nursing students who enrolled in a nursing school in Fukuoka Prefecture, Japan, in April 2018. The nursing school was established in 2017 and became the 12th nursing college in the prefecture. Therefore, there were only first‐ and second‐year nursing students in the school at baseline.

All 112 first‐year nursing students enrolled as subjects in this study. The baseline questionnaires were distributed to the 112 students after the orientation for new students in April 2018. One student did not participate in the first survey. All 111 students participated in the programmes in May 2018 and October 2018. The three oral assessment tests were conducted before and after the first education programme and after the second education programme. However, four students did not participate in the third test. The second questionnaire survey was conducted in October 2018, and six students did not participate in the second survey. Therefore, the final analysis included 101 nursing students (90.2% of the first‐year nursing students).

### 2.2 Questionnaires

To investigate the effect of interprofessional educational programmes designed for the improvement of nursing students' oral assessment performance, the questionnaire items were derived from a previously developed questionnaire for evaluating nurses' attitudes, confidence, abilities, and performance of oral assessments. The validity and reliability of the questionnaires were verified in previous studies (Haresaku et al., 2018a; Haresaku et al., 2018b). However, the items in the questionnaire regarding their oral assessment performance for their patients were excluded from this study. Cronbach's alpha values for each domain ranged from.827 to.913. Prior to the study, the questionnaire was pilot‐tested with nursing students who were 2‐year students in the nursing school. They were not included as subjects in this study.

The questionnaire consisted of the following five parts: sociodemographic data (two items), attitudes towards oral assessment and dental referral (nine items), confidence with performing oral assessment and dental referral (nine items), frequencies of the performance of self‐oral assessment (one item), and oral assessment categories of performance of self‐oral assessment (six items).

The collected sociodemographic information included gender and age. To evaluate their attitudes in relation to the performance of oral assessments and dental referrals, they were asked whether they felt that nurses could assess (a) the status of oral hygiene, (b) the presence of dental caries, (c) the presence of periodontal disease, and (d) the presence of oral cancer in their patients. Additionally, they were asked whether they felt nurses should perform (a) oral assessment and (b) dental referral. They were also asked whether they hoped they perform (a) oral assessment and (b) dental referral if they will have a qualification in the future. A 5‐point Likert scale was used for *strongly agree*, *agree*, *neither*, *disagree*, or *strongly disagree*; the attitude scores ranged from 1 (*strongly disagree*) to 5 (*strongly agree*).

To assess their confidence with the performance of oral assessment, they were asked how confident they felt examining in each oral assessment category and assessing the needs of dental referral. The oral assessment categories, which were defined based on the Oral Health Assessment Tool (OHAT), were as follows: (a) the lip (swelling, bleeding, and ulceration), (b) the tongue and tongue coating, (c) the gingiva and oral mucosa (swelling, bleeding, and ulceration), (d) saliva (quality and quantity), (e) the present teeth (decayed teeth or tooth fracture), (f) removable dentures (broken area), (g) oral cleanliness (food debris, calculus, and plaque), and (h) oral pain (verbal and/or non‐verbal signs of pain). A 5‐point Likert scale was used, allowing for confidence scores to range from 1 (*no confidence*) to 5 (*strong confidence*). The examination of the oral categories of the OHAT by nonoral professionals (nurses) was shown to be valid and reliable in a previous study (Chalmers et al., 2005).

To evaluate the frequencies of their performance of self‐oral assessment, they were asked how many times they performed self‐oral assessment per week. The frequencies were divided into five categories: (a) seldom, (b) feeling some kind of symptoms in the mouth, (c) one to three times per week, (d) four to six times per week, and (e) every day. If they chose choices except for “seldom,” they were asked whether they assessed each oral assessment category based on the OHAT excepted for removable dentures and oral pain.

### 2.3 Educational programmes

A course for oral health education in the first‐year curriculum was created by dentists in the nursing school or in a dental school that belonged to the same school cooperation. The course had eight programmes on oral health for 12 hr and one credit. The oral assessment programme in the course was performed by two dentists whose profession was preventive or geriatric dentistry, and other programmes were paediatric, adult, and geriatric dentistry. The oral assessment programme was a 1.5‐hr programme conducted in May 2018 by the dentist who belonged to the nursing school. The dentist who moved to the nursing school from a dental school in 2017 oversaw oral health care education and oral assessments at the school. The first part of the programme was lecture, which covered basic oral anatomy, the importance of oral health for general health and quality of life, the importance of collaborations among oral health care professionals to enable patients to prevent oral and systemic diseases, and the purpose of nurses' oral assessment. After the lecture finished, dental mirrors and hand mirrors were distributed to the students. They were instructed on how to assess the oral assessment categories and the needs of dental referrals using the OHAT sheet. After the instruction, they performed self‐oral assessment with the mirrors and assessed their oral assessment categories. After the first programme, there was a 1‐hr partner oral assessment training in the basic nursing course in October 2018. The nursing students were paired. One of the paired students performed an oral assessment with a dental mirror and OHAT sheets, explained the results of the oral assessment, and performed oral health care with toothbrush and dental spouge to his or her partner within 15 min. After the student finished performing the tasks, they switched partners and repeated the exercise. After both students performed the exercise, they changed partners and performed the exercise again. The programme was conducted by the dentist and the nursing academic staff in the nursing school. After the nursing students were instructed on how to assess each oral assessment category, they conducted the partner training of oral assessment with a pen light and the OHAT sheet.

### 2.4 Oral assessment ability tests

First, OHAT sheets were distributed to the students so that they could complete the category scores in each oral assessment category. The eight images of oral status used to assess each of the eight oral assessment categories were projected sequentially for 30 s on the LCD screen. During the projection of the images, they assessed the images and assigned the category scores (healthy = 0, changes = 1, or unhealthy = 2) on the sheet. After assigning the scores, they answered whether a dental referral was needed (no = 0 or yes = 1). If their scores corresponded with the dentists' scores, they were considered to be the correct answers. The total number of correct answers (0–9) and the percentages of correct answers according to the oral assessment categories were calculated. The second and third tests were conducted in the same way as the first tests.

### 2.5 Statistical analyses

A Wilcoxon signed‐rank test was used to compare the attitude scores, the confidence scores, and the total scores of the correct answers in the oral assessment ability tests before and after the educational oral assessment programmes. A chi‐square test was used to explore the differences in the distributions of the correct answers in oral assessment ability tests, the frequencies of their performance of self‐oral assessment per week, and their self‐oral assessment performance in each oral assessment category between the time before and after the programmes. Missing data were excluded from the analysis. The data were analysed at the 5% significance level. The statistical analyses were performed using the IBM SPSS Statistics software program (version 21.0; IBM Corporation, Armonk, NY, USA).

### 2.6 Ethical considerations

This study was approved by the Ethics Committee of Fukuoka Gakuen, Fukuoka, Japan (approved #375). The front page of the questionnaire explained the aim of the project and the voluntary nature of the study. The second page was the consent form. If the students agreed to participate, then they signed the consent form and completed the questionnaire. They were instructed that their participation was of their own free will and that they could leave the room without signing the consent form or completing the questionnaire if they did not want to participate in the study. After the students had signed the consent form and completed the questionnaire, they put it in a box and then left the room. Before the oral assessment tests were conducted, the students were instructed that the results of the tests did not affect their academic results and that they could put the OHAT sheet in the box without completing it and leave the room. They were also instructed that they could refuse to allow the use of their data in this study at any time.

## 3 RESULTS

A total of 101 (90.2%) first‐year nursing students participated in this study. They were all Japanese, and the majority (88.3%) were female. Almost all (96.4%) were 18–19 years old, and the others (3.6%) were 20–23 years old.

Table 1 shows the comparison of the attitude scores before and after the programmes. The average attitude scores for their feelings regarding whether nurses could assess the presence of caries, periodontal disease, and oral cancers were approximately 3 points at baseline. On the other hand, the average scores for their feelings regarding whether nurses should perform oral assessment and dental referrals and their willingness to perform dental referrals in the future were more than 4 points at baseline. All average scores, except for the average score of their willingness to perform oral assessments in the future, increased after the programmes (*p* < .05).

**Table 1:** Comparison of the attitude scores (1–5) before and after the programmes

	Baseline (average score ± *SD*)	After the programmes (average score ± *SD*)	*p* value^a^
Nurses can assess the status of oral hygiene in their patients.	3.57 ± 0.95	4.12 ± 0.91	<.001
Nurses can assess the presence of dental caries in their patients.	3.00 ± 1.00	4.04 ± 0.85	<.001
Nurses can assess the presence of periodontal disease in their patients.	3.19 ± 1.00	4.09 ± 0.85	<.001
Nurses can assess the presence of oral cancer in their patients.	3.05 ± 1.10	3.98 ± 0.95	<.001
Nurses should perform oral assessment to perform appropriate oral health care for their patients.	4.25 ± 0.77	4.44 ± 0.77	.037
Nurses should perform oral assessment to their patients and encourage their patients with dental problems to see a dentist.	4.10 ± 0.85	4.44 ± 0.70	.001
If I have a nurse qualification in the future, I hope I perform oral assessment for my patients.	3.84 ± 0.95	4.00 ± 1.04	.130
If I have a nurse qualification in the future, I hope I perform oral assessment for my patients and encourage those with dental problems to see a dentist.	4.05 ± 0.94	4.37 ± 0.86	.001
Total of the attitude scores	32.50 ± 5.54	37.17 ± 5.70	<.001

*Note.* Score 1 = *strongly disagree*, Score 2 = *disagree*, Score 3 = *neither*, Score 4 = *agree*, and Score 5 = *strongly agree.*

Abbreviation: *SD*, standard deviation.

a Wilcoxon signed‐rank test.

Table 2 shows the confidence scores before and after the programmes according to the oral assessment categories. The average confidence scores were less than 3 points in all oral assessment categories at baseline. The average score (standard deviation) for the assessment of dental referral needs was the lowest at 2.18 (0.96) points. However, the average scores increased more than 3 points in all categories (*p* < .05).

**Table 2:** Comparison of the confidence scores (1–5) before and after the programmes according to the oral assessment categories

	Baseline (average score ± *SD*)	After the programmes (average score ± *SD*)	*p* value^a^
Lip (swelling, bleeding, and ulceration)	2.75 ± 0.98	3.93 ± 0.98	<.001
Tongue and tongue coating	2.43 ± 0.96	3.77 ± 1.03	<.001
Gingiva and oral mucosa (swelling, bleeding, and ulceration)	2.40 ± 0.92	3.80 ± 1.01	<.001
Saliva (quality and quantity)	2.23 ± 1.01	3.56 ± 1.13	<.001
Present teeth (decayed or broken teeth)	2.50 ± 1.02	3.68 ± 1.05	<.001
Removable dentures (broken area)	2.70 ± 1.45	4.10 ± 0.96	<.001
Oral cleanliness (food residue, calculus, and plaque)	2.53 ± 0.97	3.81 ± 0.94	<.001
Oral pain (verbal and/or non‐verbal signs of pain)	2.68 ± 1.25	3.84 ± 1.08	<.001
Assessment of the needs of dental referral after the oral assessment performance	2.18 ± 0.96	3.75 ± 1.08	<.001
Total of the confidence scores	22.09 ± 7.32	34.25 ± 7.21	<.001

*Note.* Score 1 = *no confidence* and Score 5 = *strong confidence.*

Abbreviation: *SD*, standard deviation.

a Wilcoxon signed‐rank test.

The average total scores (standard deviation) on the first, second, and third oral assessment abilities tests were 6.80 (1.33), 6.96 (1.38), and 7.88 (1.01) points, respectively. There were significant differences in the average total scores between the first and third tests (*p* < .001), although there were no significant differences in the scores between the first and second tests (*p* = .414).

Table 3 shows the comparison of the percentages of the correct answers on oral assessment ability tests according to oral assessment categories. The percentages of correct answers were lower than 50% in the categories of lip and denture removal on the first test. However, the percentages were higher than 75% in all categories on the third test. There were significant differences in the percentages of correct answers for gingiva and oral mucosa, saliva, removable dentures, oral pain, and needs for dental referral between the first and third tests (*p* < .05).

**Table 3:** Comparison of the percentages of the correct answers in oral assessment ability tests according to oral assessment categories

	Correct answer^a^	First test (%)	Second test (%)	Third test (%)	*p* value^b^	*p* value^c^
Lip	2	80.2	60.4	78.2	.002	.729
Tongue and tongue coating	1	76.2	68.3	77.2	.209	.868
Gingiva and oral mucosa	2	46.5	74.3	80.2	<.001	<.001
Saliva	2	68.3	58.4	88.1	.144	.001
Present teeth	2	92.1	98.0	95.0	.052	.390
Removable dentures	2	47.5	65.3	82.2	.011	<.001
Oral cleanliness	2	94.1	94.1	89.1	1.000	.205
Oral pain	2	81.2	77.2	98.0	.488	<.001
Need for dental referral	1	94.1	100.0	100.0	.013	.013

a The category scores that the dentist answered.

b The first test versus the second test, chi‐square test.

c The first test versus the third test, chi‐square test.

Regarding the frequencies of the nursing students' self‐oral assessment, the majority performed self‐oral assessment when they felt symptoms in their mouth both at baseline and after the programmes (Table 4). The percentages of their performing self‐oral assessment every day increased from 15.8% at baseline to 32.7% after the programmes. There was a significant difference in their self‐oral assessment performance before and after the programmes (*p* < .05).

**Table 4:** Comparison of the nursing students' self‐oral assessment performance

	Baseline *n* (%)	After the programmes *n* (%)	*p* value^a^
How often do you perform self‐oral assessment per week?			
Seldom	8 (7.9%)	6 (5.9%)	.012
When I feel symptoms in my mouth	56 (55.4%)	36 (35.6%)	
1–3 times per week	16 (15.8%)	15 (14.9%)	
4–6 times per week	5 (5.0%)	11 (10.9%)	
Every day	16 (15.8%)	33 (32.7%)	

a Chi‐square test.

Table 5 shows nursing students' self‐oral assessment performance according to oral assessment categories. More than 60% performed self‐oral assessment in present teeth and oral cleanliness at baseline, although less than half performed the assessment in other categories. The percentages increased in all categories after the programmes, and there were significant differences in their self‐oral assessment performance of tongue, tongue coating, and saliva before and after the programmes (*p* < .05).

**Table 5:** Comparison of nursing students' self‐oral assessment performance according to oral assessment categories

	Baseline *n* (%)	After the programmes *n* (%)	*p* value^a^
Lip (swelling, bleeding, and ulceration)	41 (40.6%)	45 (44.6%)	.569
Tongue and tongue coating	25 (24.8%)	45 (44.6%)	.003
Gingiva and oral mucosa (swelling, bleeding, and ulceration)	43 (42.6%)	54 (53.5%)	.121
Saliva (quality and quantity)	1 (1.0%)	7 (6.9%)	.030
Present teeth (decayed or broken teeth)	64 (63.4%)	74 (73.3%)	.130
Oral cleanliness (food residue, calculus, and plaque)	61 (60.4%)	71 (70.3%)	.139

a Chi‐square test.

## 4 DISCUSSION

This report is the first to investigate the effect of interprofessional educational programmes that specialize in improving nursing students' attitudes, confidence, abilities, and performance of oral assessments. Approximately 90% of nursing students were female, and almost all (96.4%) were under 19 years old. The total percentage of males and females in Japanese nursing schools was 10.3% and 89.7%, respectively, in 2016 (Ministry of Education, Culture, Sports, Science and Technology, 2017). The percentage of first‐year students aged 18–19 years in Japanese nursing schools was 96.5% (Kashiwada, 2017). Therefore, it seemed that the distributions of those characteristics in this nursing school were normal compared with other Japanese nursing schools.

The students' attitudes regarding nurses being able to assess oral health were not positive at baseline. However, attitudes improved to a somewhat positive level after the interprofessional educational programmes. A previous study of oral assessment education for 55 nurses in a hospital reported that the programmes that combined lectures and partner training were effective in improving attitudes towards oral assessment administration (Haresaku et al., 2018b). In addition, the nurses' attitudes regarding their duties and their willingness to perform oral assessments and dental referrals improved after the programmes, even if their attitudes were somewhat positive at baseline. These positive attitudes may contribute to their performance of oral assessments and dental referrals for their patients in the future.

The students' confidence in oral assessment performance improved in all assessment categories after the educational programmes; therefore, these programmes might be effective in facilitating performance improvement. A previous study of an interprofessional oral health assessment educational programme, in which dental faculty and students taught nursing students, reported that the students' confidence in performing oral assessments improved after the programmes (Estes et al., 2018). On the other hand, the educational programmes for oral assessment offered for nurses were not effective in improving the nurses' confidence (Haresaku et al., 2018b). Therefore, it is suggested that oral assessment educational programmes should be conducted for nursing students before they become nurses so as to improve their confidence in performing oral assessments.

Some previous studies regarding oral health interprofessional education for nursing students reported that students' attitudes and confidence regarding oral health improved after the education intervention (Cooper et al., 2017; Golinveaux et al., 2013). Therefore, interprofessional education with dental and nursing staff may be effective for improvement of attitude and confidence.

There have been few studies regarding oral assessment abilities among nursing students, although some studies of nurses have shown that the validity and reliability of oral assessments were improved after the education intervention (Andersson et al., 2002; Aoki et al., 2018; Chalmers et al., 2005; Eilers et al., 1988; Tsukada et al., 2017). The oral assessment abilities among the nursing students in this study were measured by the total scores or the percentages of correct answers. The average total scores did not improve after only the first programme; rather, the scores significantly improved after the second programme. These results indicated that not only the lecture on oral assessment with self‐oral assessment but also the partner training on oral assessment might be needed to improve students' oral assessment abilities. The ability to assess the lips, tongue, and tongue coating did not improve. On the other hand, the percentage of correct answers regarding gingiva and oral mucosa and removable dentures significantly increased from less than half at baseline to more than 80% after the programmes. Thus, their oral assessment abilities of assessing gingiva and oral mucosa and removable denture might be improved easily.

The frequencies of their self‐oral assessment performances per week improved, and the percentage of their performance of daily self‐oral assessments more than doubled; therefore, the education programmes might be effective in improving students' self‐oral assessment performance. The improved oral assessment categories were the tongue, tongue coating, and saliva. A previous study regarding oral health interests among young Japanese women reported that they paid more attention to the smell of the breath and caries compared with a variety of other oral conditions (Mineoka et al., 2011). The nursing students in this study were informed in the programmes that the tongue coating is one of the main indicators of halitosis and that saliva is important for caries prevention. Therefore, the students might be interested in the prevention of halitosis and dental caries, and the knowledge they acquired in the programmes might affect their improvement in performing self‐oral assessment of the tongue coating and saliva. A previous study reported that the programme of self‐assessment and subsequent faculty feedback enabled dental students to improve their performance on technical assessments (Deeb, Carrico, Laskin, & Koertge, 2019). Therefore, our students' improvement in performing self‐oral assessments might positively affect their oral assessment abilities.

Several limitations associated with this study warrant mention. The subjects included only 112 first‐year nursing students in a Japanese nursing school. Therefore, all the nursing students in the school were included in this study without regard to a power calculation. The data in this study were from a nursing school in Fukuoka Prefecture in Japan. The number of Japanese nursing schools (4‐year schools) was 267 in 2017 (Japanese Nursing Association, 2015). Therefore, there would likely be variation of characteristics among the schools. However, the distributions of the characteristics in the nursing school were normal; therefore, they were unlikely to affect the results in this study.

The control group was not included in this study. All of the students needed to take these programmes at the same time, and the oral educational programmes have been included in the curricula since the school opened in 2017. Therefore, it was impossible to divide the sample into an intervention group and a control group with 1‐year nursing students or 1‐year nursing students with programmes versus 2‐year nursing students without programmes. Oral assessment abilities were not measured by oral assessment of real patients but rather were measured using images of patients. Ethical problems would arise if patients were used for the measurement of oral assessment abilities by nursing students. Therefore, further studies are needed to develop tools that enable the measurement of nursing students' oral assessment ability more effectively. The nursing students took other courses in oral health during the study period. Those courses might have affected the students' attitudes, abilities, and self‐performance regarding oral assessment and are therefore possible confounding factors in this study.

## 5 CONCLUSION

Despite the limitations of the present study, we conclude that the interprofessional education effectively improved students' attitudes and confidence in oral assessment, their oral assessment abilities, and self‐oral assessment performance. However, our study focused on their improvements in oral assessment abilities, and whether these improvements contributed to appropriate referrals is not known. Therefore, further research and programmes must focus on training to improve oral health care referrals.

## CONFLICT OF INTERESTS

All the authors have no conflict of interests to declare.
